# Regulation of MHC I Molecules in Glioblastoma Cells and the Sensitizing of NK Cells

**DOI:** 10.3390/ph14030236

**Published:** 2021-03-08

**Authors:** Timo Burster, Fabian Gärtner, Christiane Bulach, Anuar Zhanapiya, Adrian Gihring, Uwe Knippschild

**Affiliations:** 1 Department of Biology, School of Sciences and Humanities, Nazarbayev University, Kabanbay Batyr Ave. 53, 010000 Nur-Sultan, Kazakhstan; anuar.zhanapiya@nu.edu.kz; 2 Department of General and Visceral Surgery, Surgery Center, Ulm University Hospital, Albert-Einstein-Allee 23, 89081 Ulm, Germany; fabiangaertner@hotmail.de (F.G.); Christiane.Bulach@outlook.com (C.B.); Adrian.gihring@uni-ulm.de (A.G.); uwe.knippschild@uniklinik-ulm.de (U.K.)

**Keywords:** proteases, NK cells, MHC, cathepsin G, immunotherapy

## Abstract

Immunotherapy has been established as an important area in the therapy of malignant diseases. Immunogenicity sufficient for immune recognition and subsequent elimination can be bypassed by tumors through altered and/or reduced expression levels of major histocompatibility complex class I (MHC I) molecules. Natural killer (NK) cells can eliminate tumor cells in a MHC I antigen presentation-independent manner by an array of activating and inhibitory receptors, which are promising candidates for immunotherapy. Here we summarize the latest findings in recognizing and regulating MHC I molecules that affect NK cell surveillance of glioblastoma cells.

## 1. Introduction

The choice of cancer treatment depends on the type of tumor and its progression, as well as the age, comorbidity, and compliance of the patient. Often a combination of treatments is considered, such as surgery combined with chemotherapy and/or radiotherapy. For many patients, surgery is the chosen form of treatment, which is applied for solid tumors. In order to be successful, surgery must ensure that the cancerous tissue is completely removed. Aside from early diagnosis and treatment, some tumor cells can remain in the body, leading to recurrence of the tumor. These cells are known as cancer stem cells (CSCs). CSCs are tumor-initiation cells, since they represent a subpopulation of tumor cells with stem cell-like characteristics, involving proliferation, differentiation, and self-renewal. Therefore, it is crucial to eliminate these CSCs with a therapeutically useful approach to avoid a relapse [1].

Astrocytoma grade IV (glioblastoma) is one of the most aggressive tumors of the central nervous system and is characterized by high vascularity, fast proliferation, and diffuse invasion of glioblastoma cells into the surrounding healthy tissue. The present therapeutic treatment comprises surgical removal of the tumor followed by radio- and chemotherapy, with the latter using the standard chemotherapeutic drug temozolomide, which prolongs survival and quality of life for only a few months [2,3]. Glioblastoma cells and glioblastoma stem cells are highly resistant to treatment. Even though several novel treatment strategies have been established, more effective and more tolerable therapies are needed, particularly for patients at an advanced age. An encouraging anti-tumor strategy is immunotherapy, which includes a variety of therapeutic options by taking advantage of the immune system, such as application of monoclonal antibodies, immunomodulatory components, chimeric antigen receptor (CAR) T cells, the CAR natural killer (NK) cell, or combined treatment with NK cells and drugs [4,5,6,7].

In particular, NK cells are considered to be promising candidates in the field of immunotherapy. Furthermore, this review will focus on mechanisms that are associated with the interaction of inhibitory receptors on NK cells with major histocompatibility complex class I (MHC I) molecules and will summarize the regulation of MHC I, the distribution of inhibitory molecules on NK cell subsets, as well as the interaction of those molecules with MHC I on tumor cells.

## 2. Defective Antigen Presentation of Tumor Cells Is a Result of Immune Evasion

At an early stage, immune effector cells can eliminate nascent transformed cells. Intensive and invasive growth of the tumor can lead to minor disruptions of the surrounding tissue by induction of inflammatory processes, which can be monitored by the recruitment of immune cells, such as NK cells, dendritic cells (DCs), or macrophages followed by the infiltration of tumor-specific CD4^+^ and CD8^+^ T cells to the tumor site in order to attack the remaining antigen-bearing tumor cells [8]. As a result, interactions between the tumor and the immune system take place, creating a selective pressure in the tumor microenvironment (TME), finally leading to a malignant progression or a successful elimination of tumor cells by the immune system [9].

The antigen (antigenic peptide) binding protein MHC I (major histocompatibility complex class I) is assembled by a heavy chain (α1, α2, and α3) attached to β2-microglobulin and transported to the cell surface bearing the antigen. CD8^+^ T cells monitor these peptides bound to MHC I molecules, compared to CD4^+^ T cells which distinguish antigenic peptides bound to MHC II molecules; these are normally presented by professional antigen presenting cells (APCs). MHC I molecules are encoded by the three classical loci including human leukocyte antigen A (HLA-A), HLA-B, and HLA-C, which are related to the non-classical HLA molecules (HLA-E, HLA-F, and HLA-G) [10]. Like normal cells, tumor cells present antigens on the cell surface via MHC I, which can be tumor-specific- or tumor-associated antigens. The antigen-MHC I-complex is encountered by the T cell receptor (TCR) of CD8^+^ T cells; however, tumor cells may lose the ability to express cell surface MHC I molecules by intracellular arrest of MHC I or by acquiring defects in the antigen presentation pathways. Consequently, CD8^+^ T cells cannot recognize tumor cells and evade immune recognition [11]. This would be the case if there are no alternative paths for the immune system to eliminate tumor cells. Thus, NK cells attack cells lacking sufficient MHC I cell surface expression, which is also called missing self-recognition. In order to circumvent NK cell recognition and elimination, tumor cells encompass an additional immune evasion repertoire by instantly maintaining the presence of a limited set of cell surface MHC I molecules and escaping surveillance by NK cells [12].

Additionally, tumor cells have developed further mechanisms to prevent recognition and destruction by NK cells. An important factor is the tumor environment. The conditioning of suppressive cells, such as T regulatory cells (Tregs), M2 macrophages, and myeloid-derived suppressor cells (MDSCs) favor a suppressive environment leading to tumor immune evasion [13]. Additionally, hypoxia, which frequently occurs in tumors, leads to the inhibition of NK cell function due to the down-regulation of activating receptors on NK cells. The decrease of activating ligands on tumor cells, including natural killer group 2D ligands (NKG2DL), which can bind to the natural killer group 2D activating receptor (NKG2D) of NK cells, results in tumor escape from NK cell detection. NKG2DL, like the MHC class I chain-related protein A and B (MICA and MICB) or the UL 16 binding protein 1–6 (ULBP1–6), are highly expressed on the surface of stressed cells but also by tumor cells [14]. Interestingly, tumor cells are able to shed MICA and MICB from their surface [15], which can bind to the cell surface NKG2D and provokes an internalization of NKG2D into NK cells [16]. The reduction of cell surface NKG2D, as a result of internalization without activation of NK cells, is referred to as a desensitization of NK cells and represents an additional immune evasion strategy of tumor cells [17,18]. NK cells are activated when MHC I molecules are missing on the cell surface. Therefore, the expression pattern of MHC I molecules on gliomas, including glioblastoma cells, might be highly relevant for the use of NK cells for treatment. For instance, glioma stem cells, isolated from surgical samples, have been shown to downregulate MHC I molecules associated with an activation of the Wnt/β-catenin signaling pathway leading to evasion from CD8^+^ T cells [19,20]. NK cells can be recruited from the blood and cross the blood-brain barrier via the CXXXCL1 gradient secreted by neurons or CCL2 and CXCL10 released by microglia and astrocytes under inflammatory conditions [21]. Thereby, NK cells might be able to target glioma stem cells. However, the downregulation of MHC I is not generally true for all gliomas and is controversially described in the literature. Generally, reduced levels of MHC I depend on the patient sample or cell lines investigated in the assays [22]. In the next section, we will discuss the different NK cell subsets and their function.

## 3. Biology of NK Cell Subsets

Antibodies are divided into two domains, the Fab and Fc. While the Fab region of antibodies binds to a distinct antigen epitope, the Fc region is recognized by the Fc receptor. The Fc domain of IgG binds to FcγIII (CD16) which is found on NK cells and on immune cells of the innate and adaptive immune system. Simultaneous binding of cell-surface tumor antigens and CD16 of NK cells triggers antibody-dependent cell-mediated cytotoxicity (ADCC). The antibody forms a bridge between the tumor cell and the NK cell and generally enables NK cells to eradicate tumor cells [23]. A special feature of CD16 represents its ability to induce cytolytic activity of NK cells without the support of other costimulatory molecules. As a result of NK cell activation, NK cells secrete several cytokines, interferon (IFN)-γ, tumor necrosis factor-α (TNF-α), granulocyte macrophage colony-stimulating factor (GM-CSF), and chemokines (chemokine C-C motif ligand 1, CCL1, CCL2, CCL3, CCL4, CCL5, and CXCL8), thereby modulating the function of the innate and adaptive immune system. The release of cytoplasmic granules, containing perforin and granzymes, and the expression of death receptor ligands, such as FasL and TNF-related apoptosis-inducing ligand (TRAIL), can trigger apoptosis in target cells [12].

The identification of classical NK cells and their subsets has been well established over the last decades. Conventional markers used to determine NK cell subsets are CD3, CD56, and CD16 [24]. In the first approximation, NK cells can be defined by cell size and the missing expression of CD3. The cell-surface density of CD16 and CD56 can mainly be used for further characterization of NK cells into regulatory and cytotoxic subsets. The CD56^+^CD16^™^ NK cell subset has a regulatory capacity, CD56^+^CD16^+^ NK cells can be cytotoxic to target cells, and CD56^™^CD16^+^ NK cells are regarded as a kind of “defective” subset among NK cells, which have increased numbers of activating and inhibitory receptors but are deficient in their ability to be cytolytic. These cells are also impaired in cytokine production and proliferation [25,26].

Recent developments in the field of flow cytometry and phenotyping approaches by using mass cytometry by time-of-flight (CyTOF) have created a high possibility for deep profiling of NK cells. The simultaneous detection of the lysosomal marker CD107a, perforin to differentiate between newly synthesized and lytic granule-associated perforin, granzyme B, cytokines, proteolytically active proteases, and cell surface markers leads to a high-resolution phenotyping of NK cell biology [27,28,29,30]. Evidence can be gained from activation signals, like NKG2D and NKp46, which play crucial roles in anti-tumor immunity [31,32]. Moreover, several markers are used to describe the development of NK cells, among them CD27, CD57, CD62L, and CD127 [33,34], whereas CD57 is specifically used for the identification of terminally differentiated NK cells. For instance, CD57 is not expressed on NK cells from newborns, but the density of cell surface CD57 increases with age and correlates with the maturation process of NK cells, which thus have an impaired proliferation capability and increased expression of killer-cell immunoglobulin-like receptors (KIRs) [35]. Although surface markers can be used to categorize and characterize NK cells, activating and inhibitory receptors are crucial in determining the function and specific role of NK cells in the immune system and will be addressed in the following paragraph.

### 3.1. Activating and Inhibitory Receptors of NK Cells

In order to be able to recognize tumor cells, NK cells possess different surface receptors. The net sum of signals through these receptors determines the behavior of NK cells. These receptors are roughly divided into activating and inhibitory receptors and can recognize altered expression of self-proteins on target cells. Tumor cells have developed strategies to bypass NK cell activation by attenuating activating receptors or by stimulating inhibitory receptors [36]. The activating receptor binds to ligands whose expression is restricted to infected and transformed cells. These include NKG2D, DNAX accessory molecule 1 (DNAM-1), and the natural cytotoxicity receptors (NCR), such as p30, p44, and p46-related proteins (NKp30, NKp44, and NKp46). However, NKp44 is only expressed on previously activated NK cells and downregulation of NCRs correlates with lower cytolytic activity. Specifically, NKG2D plays a crucial role in the activation of NK cells by binding to MICA, MICB, and ULBP 1–6. In contrast to normal cells, NKG2D ligands are rarely expressed to avoid autoimmunity [37].

Transformed cells, but also virus-infected cells, down-regulate or alter the expression of cell surface MHC I molecules on tumor cells, which restricts CD8^+^ T cells in the recognition of tumor antigens. NK cells, on the other hand, express cell surface inhibitory receptors to specifically recognize MHC I molecules and inspect levels of MHC I which differ between normal and transformed cells. In other words, when MHC I molecules are absent, the inhibitory receptors discontinue the inhibition of the intracellular cascade of the activating receptor and provokes activation of NK cells [38,39]. Immature NK cells express the inhibitory receptor CD94:NKG2A heterodimer that binds specifically to HLA-E [40]. In comparison, late stage differentiated NK cells dominantly harbor the inhibitory receptors killer-cell immunoglobulin-like receptors (KIRs) on the cell surface [39,41]. However, the KIR receptor family represents members that have either inhibitory or activating functions. Hereby, the inhibitory KIRs show a higher avidity for MHC I compared to activating KIRs [42]. Activating and inhibitory function is in general based on the length of the cytoplasmic domain. KIRs with long cytoplasmic domains, except for KIR2DL4, show inhibitory responses including the recruitment of inhibitory protein tyrosine phosphatases in contrast to short cytoplasmic domains. Signal transduction of KIRs is mediated through an association with immunoreceptor tyrosine-based activation motif (ITAM), present in the cytoplasmic region of the DAP12 molecule [43]. Furthermore, KIRs are defined by their numbers of extracellular C2-type Ig-like domains. KIR2D contains two extracellular domains (D1 and D2) and KIR3D contains three, referred to as D0, D1, and D2 [44,45]. While ligands and functions of inhibitory KIRs are well defined and will be discussed later, a detailed classification is missing for activating KIRs. Chewning et al. showed that activating KIRs respond well when encountering allogeneic MHC I molecules [46]. Furthermore, NK cells expressing the activating molecule KIR2DS2 kill glioblastoma cells very efficiently in mice [47]. The expression of various inhibitory and activating receptors on NK cells can be linked with the function of the NK cell subset.

### 3.2. NK Cell Subsets and Their Function

The expression of KIR and its interaction with MHC I molecules differs between various subsets of NK cells. CD56^bright^ NK cells show low levels of KIR or even lack KIR completely. These subsets show a decreased cytotoxic activity in ex vivo experiments and can be found in the lymph nodes and the secondary lymphoid tissue. This is in contrast to CD56^dim^CD16^bright^ NK cells expressing a high amount of KIR and are predominantly found in the peripheral blood with high levels of baseline perforin [48,49].

Immature NK cells harbor NKG2A which binds with high affinity to HLA-E and is expressed early during NK cell maturation [40]. Further maturation leads to the acquisition and co-expression of KIRs, thereby discontinuing NKG2A expression, and mainly KIR inhibitory receptors are found on mature NK cells [50,51,52]. In order to avoid autoreactivity, all mature NK subsets express at least one kind of inhibitory receptor that can detect self-MHC I molecules [53]. One exception of cell surface expression of KIR is due to unlicensed NK cells, since unlicensed NK cells lack the expression of inhibitory self-receptors per definition [54]. In the next section, we will focus on how inhibitory molecules and MHC molecules interact on the molecular level.

### 3.3. Molecular Interaction of the Inhibitory Receptor with MHC I

NKG2A, a heterodimer complex of CD94-NKG2A, associates with HLA-E and binds to the C-terminal end of the antigen-binding cleft at an angle of around 70°. Petrie and colleagues showed, by analyzing the crystal structure of CD94-NKG2A and HLA-E, the specific interactions between these molecules. Thereby, CD94 attaches to the α1 domain, and NKG2A to the α2 of HLA-E; α1 and α2 generate the antigen-binding groove of HLA-E. This interaction is mainly directed by electrostatic interaction based on charge complementarity. However, CD94 association is also influenced by hydrogen bonds (Gln113 to Asp69, Asn170/Glu164 to Gln72, Thr146 to Glu89, and Ser143 to Arg79 of HLA-E), hydrophobic interactions (Phe114 to Ile73 and Leu162 to Val76 of HLA-E), and salt bridges (Arg171 to Asp69 and Asp163 to Arg75 of HLA-E). The crucial interaction between NKG2A and HLA-E are based on hydrogen bonds (Arg137 to Ser151 and Ser172 to His155 of HLA-E), salt bridges (Arg137 to Glu154 and Lys217 to Asp162 of HLA-E), and Van der Waals forces (Pro171 to His155 and Gln212/Lys217 to Ala158 of HLA-E) [55]. This interaction does not provoke a conformation change and was observed previously in thermodynamic studies [56].

KIRs with long cytoplasmic domains can interact with HLA-A, -B, or -C resulting in inhibition (except for KIR2DL4). HLA-A and HLA-B are recognized by KIR3D, while HLA-C binds to KIR2D [45]. The interaction between the highly polymorphic KIR family and HLA isotypes was previously described for KIR2D, which associates to HLA-C, as well as KIR3D and its association to HLA-B. The binding of KIR2D to HLA-C was determined by analyzing the crystal structure of KIR2DL2-HLA-Cw3 [57] and KIR2DL1-HLA-Cw4 [58]. KIR2D binds HLA-C in an orthogonal orientation with six loops from D1 and D2 at the α1 and α2 helix, directly contacting the peptide at positions 7 and 8 without leading to any significant conformational changes. The interface of the KIR/HLA interaction is dominated by a complementary charge, while disrupted salt bridges can diminish the binding. Differences between KIR2DL2 and KIR2DL1 can explain the allotype specificity. An intermolecular hydrogen bond participates between Lys44 (KIR2DL2) and Asn80 (HLA-Cw3), while KIR2DL1 hosts the Lys80 residue in a pocket specific to KIR2DL1 and enables interaction with HLA-C [59,60]. Furthermore, Vivian and colleagues revealed the interaction of KIR3DL1 with HLA-B*5701 by analyzing the crystal structure and reported that the contact between both molecules is a two-step process. The D0 domain acts as an innate sensor by binding a highly preserved region of HLA-B (β2-microglobulin) with limited polymorphism. The second contact is made by D1 and D2. The interaction of D2-HLA-B*5701 is mainly driven by “complementarity”, while the D1 contacts with HLA-B*5701 are suboptimal due to sequence variation of the polymorphic region of HLA-B [61].

Differences of KIRs and their subsequent inhibition or activation explain the function of various NK cell subsets. Such observations can be obtained from unlicensed NK. Unlicensed NK cells are dependent on an activating ligand to stimulate the NK response and are triggered as soon as an activating ligand is expressed on the target cell which makes unlicensed NK cells potent protectors during viral infections or for targeting tumor cells [62,63].

## 4. NK Cells as a Possible Immunotherapy Approach to Treat Glioblastoma

Although it has been shown that NK cells can successfully inhibit systemic metastasis of glioblastoma in a mouse model [64], the focus of NK cells to be assessed against glioblastoma is generally based on therapies affecting the tumor within the brain. Before discussing the abilities of NK cells for immunotherapy approaches, the question remains as to how NK cells migrate across the blood brain barrier and infiltrate the glioblastoma. In addition, only 2% of immune cells infiltrating glioblastoma are NK cells [65], NK cells follow a CX3CL1 gradient to cross the blood brain barrier by expression of CX3CR1 [66]. CX3CL1 is one of the chemokines with important anti-tumoral functions [67]. Additionally, tumor-targeted polymer-conjugated checkpoint inhibitors can be applied to increase the migration of NK cells, demonstrating an effective treatment of glioblastoma by initiating a systemic and local anti-tumor immune response [68]. Successful migration of NK cells to the site of the tumor is one important part for immunotherapy, further challenges have to be addressed for an efficacious treatment option and will be described in detail in the next paragraph.

**Figure 1:**
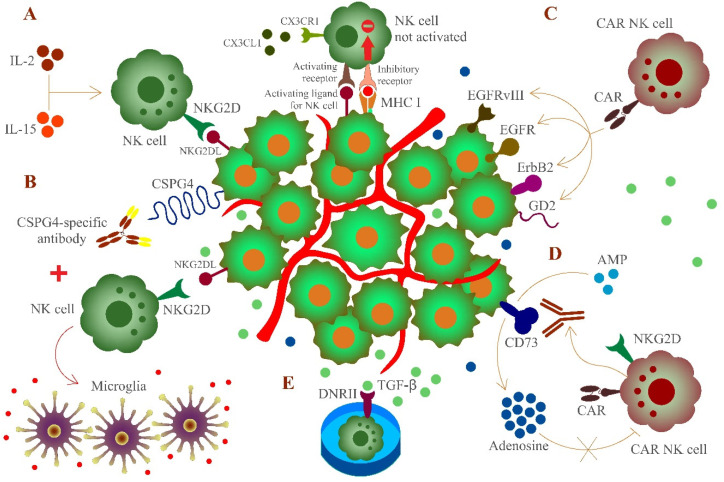
Targeting of glioblastoma cells expressing major histocompatibility complex class I (MHC I) molecules by activated or engineered natural killer (NK) cells. The chemokine receptor CX3CR1 is expressed on NK cells. NK cells can follow a CX3CL1 gradient to cross the blood brain barrier to the site of glioblastoma. However, glioblastoma cells partly express MHC I molecules to impair recognition by NK cells as an immune evasion strategy. To circumvent this obstacle in NK cell-mediated immunotherapy, several procedures were developed. (**A**) Induced upregulation of NKG2D or cytokine (IL-2 and IL-15) -activated NK cells can overcome immune surveillance, (**B**) combined use of activated NK cells and monoclonal antibodies recognizing CSPG-4 leads to the recruitment of macrophages and differentiate to microglia, provoking a proinflammatory environment, (**C**) chimeric antigen receptor (CAR)-engineered NK cells can target epidermal growth factor receptor variant III (EGFRvIII), epidermal growth factor receptor (EGFR), ErbB2, or disialoganglioside GD2 on glioblastoma cells, (**D**) engineered NK cells expressing the GD2- and NKG2D-based CARs can locally release an antibody fragment which blocks the activity of CD73 and decreases adenosine concentration, which in turn is not able to inhibit NK cell function, and (**E**) umbilical cord blood (UCB)-derived NK cells expressing the TGF-β-dominant-negative receptor II (DNRII) that is able to consume TGF-β molecules, thereby counteracting the immune-suppressive tumor environment of glioblastoma. MHC I molecules and inhibitory receptors are not shown for all cells to improve the overview.

The breakdown of immune surveillance, which might be the result of sustained immunological pressure on tumor cells developing mechanisms to become invisible to immune cells, is a critical point of tumor cells to escape immune detection [38]. Indeed, the microenvironment of glioblastoma is immunosuppressive and shows poor responses to immunotherapy [69]. This is a major challenge in developing approaches to support the immune system in combating glioblastoma cells. The engagement of NK cells for immunotherapy is very encouraging (Figure 1). However, the immunosuppressive environment and resident cells as well as the relative expression of MHC I molecules, particularly HLA-E, on gliomas have a strongly inhibitory function for NK cells [65,70,71]. Although various studies have shown that NK cells efficiently combat tumor cells [72,73,74], untreated or freshly isolated NK cells might not be sufficient to attack glioblastoma cells [71,75,76]. Instead, upregulation of activating molecules like NKG2D on NK cells [77] or the use of allogeneic and autologous cytokine-activated (IL-2 or IL-15) NK cells are important to prime NK cells to eliminate glioblastoma cells and overcome the failure of immune surveillance. In particular, utilizing NK cells activated with HSP70 and IL-2 is promising since these cells can cross the blood brain barrier and infiltrate to the site of glioblastoma [78].

The heterogeneity of tumor-associated antigens expressed on glioblastoma cells is a major obstacle for an effective immunotherapy which indicates the significance of searching for adequate markers. Previous studies indicated that chondroitin sulfate proteoglycan 4 (CSPG4, also known as NG2) a surface type I transmembrane core proteoglycan [79] being involved in several pathways regulating cell survival, angiogenesis, and migration [80], is highly expressed on glioblastoma cells (67%) with restricted intratumoral heterogeneity [81]. Long-term activation of NK cells, combined with monoclonal antibodies recognizing specific cell surface markers on glioblastoma cells, can improve a NK cell response and making them less susceptible to the tumor microenvironment. Recent evidence suggests that a combination of activated NK cells and a monoclonal antibody (mAb9.2.27), which targets CSPG4, is an encouraging strategy [82]. Moreover, the combined treatment of activated NK cells and mAb9.2.27 led to a recruitment of ED1^+^CCR2^low^ macrophages, followed by differentiation to ED1^+^ED2^low^MHC II^+^ microglia secreting pro-inflammatory cytokines [82]. However, this strategy does not bypass the issue of expression of MHC I molecules on glioblastoma cells and the subsequent inhibition of NK cells. Yet, such a combined treatment induces NK cells to create a pro-inflammatory environment and improves the survival rate of glioblastoma patients [83] by recruiting immune cells [84].

Aside from joint treatment with NK cells and antibodies, more studies of CAR-engineered NK cells to attack glioblastoma cells were designed to interfere with epidermal growth factor receptor variant III (EGFRvIII) [85], EGFRvIII and epidermal growth factor receptor (EGFR), which increased cytotoxicity and IFN-γ secretion [86], EGFR and ErbB2 [87], or disialoganglioside GD2 [88]. On the other hand, CAR-NK cell strategies have been constrained to the application of NK cell lines while showing a minimal and unreliable overall response [86,89,90], which is based on the fact that heterogeneity and diversity of glioblastoma are accompanied by various genetic and epigenetic manifestations [91]. Thus, novel treatment strategies are indicated for clinical needs, amongst them CD73, an ecto-5′-nucleotidase that has been reported to be associated with a negative survival prognosis of patients, which emerged as an effective approach to combating cancer [92]. The activity of CD73 can provoke an immunometabolic dysregulation of NK cell function by the accumulation of extracellular adenosine [93]. Glioblastoma cells expressing disialoganglioside GD2 and ligands binding to NKG2D are attacked by engineered NK cells expressing the GD2- and NKG2D-based CARs, which then locally release an antibody fragment blocking the activity of CD73 to decrease the concentration of adenosine. This combination addresses several aspects of tumor immune evasion outcomes, namely tumor antigen heterogeneity, the immunosuppressive environment, and insufficient intratumoral NK cell presence (https://www.biorxiv.org/content/10.1101/2020.10.07.330043v1, accessed on 1 March 2021). This procedure is applicable to other markers that are pro-tumorigenic, such as CD155 [94], and might offer possible treatment strategies for further investigations.

In general, the advantage of CAR-NK cells is that NK cells can be administered from a different donor to the respective recipient even with an HLA-mismatch [95]. It is possible to adopt NK cells from any blood donor to apply these cells in CAR-NK cell therapy, which makes CAR-NK cells a cost-effective treatment option without the barriers of an activation state or HLA matching [96]. An essential element for the procedure of CAR-engineered NK cells for therapy is the availability of NK cells, which is one reason for the increasing interest in assessing umbilical cord blood (UCB)-derived NK cells [97]. Additionally, the use of UCB is an improvement since the proportion of NK cells is much higher; while peripheral blood contains around 15 % NK cells of lymphocytes in contrast to UCB having about 25 % NK cells of lymphocytes [98]. Approaches with proliferative cytokines like IL-2 and IL-15 combined with genetically engineered feeder cells enable an effective expansion of UCB-derived NK cells in vitro and maintaining their cytotoxic activity [99,100]. Another strategy encompasses the application of UCB-derived NK cells expressing the TGF-β-dominant-negative receptor II (DNRII) which can consume TGF-β molecules to reduce the immune-suppressive capacity of TGF-β in the tumor environment of glioblastoma [101]. Thus, combining accessible NK cells with glioblastoma targeted approaches is a promising therapeutical tool.

## 5. Proteolytic Regulation of MHC I Molecules

It is common that cell surface receptors are internalized and recycled back to the cell surface. Internalization of MHC I from the cell surface is constitutively performed to reach the endocytic compartment for degradation or recycled back to the cell surface to present a new round of antigenic peptides to T cells [102]. Several recycling pathways (comprehensively summarized in [103]) are proposed for MHC I molecules, which are internalized to a so-called early sorting endosome in non-immune cells. First, MHC I molecules transit the early sorting endosome before being driven straight back to the cell surface, a process called the “fast recycling” pathway. Second, MHC I molecules from the early sorting endosome are directed to the trans-Golgi network (TGN) to consequently reach the cell surface. This process is termed the “retrograde transport” pathway. “Slow recycling” is the third pathway where MHC I molecules from the early sorting endosome are trafficked to the endocytic recycling compartment and then to the cell surface. The fourth pathway postulates that MHC I molecules move from the early sorting endosome to the endocytic recycling compartment followed by being maneuvered to the so-called tubular recycling endosome and finally appear at the cell surface. Another possibility is that MHC I molecules from early sorting endosomes are forced to late endosomes and then to the endocytic recycling compartment or are possibly degraded in late endosomes or lysosomes [103]. The proteolytic environment in lysosomes is responsible for the turnover of MHC I molecules [104]. It was postulated that cysteine proteases degrade MHC I molecules since the cysteine protease inhibitor E64 impaired the decrease of MHC I molecules in the endosomal/lysosomal compartment [105].

Cathepsin G (CatG), which harbors serine amino acid residue at the catalytic center (serine protease) [106], CatD (aspartic protease), and CatS (cysteine protease) proteolytically digest MHC I molecules in vitro [107]. In order to rescue MHC I from degradation, we treated peripheral blood mononuclear cells (PBMCs) with cell permeable protease inhibitors including the selective inhibitor for CatG, cysteine protease inhibitor E64d, or the aspartic protease inhibitor pepstatin A and levels of cell surface expression of MHC I were determined. The CatG inhibitor increased levels of cell surface MHC I molecules, but neither E64d nor pepstatin A altered the amount of cell surface MHC I molecules [107]. In general, human embryonic kidney 293 cells, glioblastoma cell line U87, or glioblastoma stem cells do not harbor CatG, which is suitable for insertion of CatG into the cell in order to analyze MHC I processing. Therefore, HIV Nef (Nef), which is known to provoke an internalization of cell surface MHC I [108], and CatG were overexpressed in these cells. Both CatG and Nef induced a significant downregulation of cell surface MHC I molecules. Intracellular breakdown of MHC I molecules occurred only with overexpression of CatG, but not when Nef was introduced to the respective cells because an accumulation of intracellular MHC I molecules was detected [107]. These findings suggest that CatG degrades MHC I intracellularly and Nef-driven internalization of MHC I molecules accumulate in the cell. Whether such a reduction of cell surface MHC I molecules in glioblastoma cells become a target of NK cells remains unsolved.

Based on these data, might it be possible to use CatG as a therapeutic reagent for NK cell immunotherapy? Serine proteases are commercially used to treat sepsis, hemophilia, stroke, or muscle spasms [109]. Neutrophils, when activated, release neutrophil serine proteases (NSPs), including CatG, and additional mediators at the site of inflammation. However, dysregulated proteolytic activity is dependable for many complications, such as chronic inflammatory disorders and cardiovascular diseases [106]. Moreover, it was shown that the extent of infiltrating neutrophils correlates with a severe clinical outcome of glioblastoma [110] and neutrophilia (increase of neutrophil count) is a poor prognosis of glioblastoma patients undergoing chemoradiation [111]. In general, a high level of CatG correlates with tumor grade and favors tumor progression [112]. Thus, CatG is not suitable to be delivered to the surrounding glioblastoma area.

Nanoparticles are promising delivery agents [113] also in glioblastoma [68,114]. One might speculate that CatG-loaded magnetic mesoporous silica nanoparticles (MMSN) could be delivered into glioblastoma cells to reduce MHC I molecules and sensitizing NK cells. Interestingly, neutrophils can be used as a “Trojan horse” by using immune cells as a “living” drug delivery vehicle. Thereby, neutrophils are loaded with chemotherapeutic agents and can transport the content to the site of glioma, since neutrophils can cross the blood brain barrier and infiltrate the tumor [115]. Neutrophils internalize doxorubicin-loaded MMSN (D-MMSN), whereby doxorubicin is a model anti-cancer drug, Fe_3_O_2_ for contrast in MRI, and the mesoporous silica shell is important for the encapsulation and sustained release of the chemotherapeutic agent doxorubicin. After injection of D-MMSN-loaded neutrophils to an animal model, neutrophils are attracted to the site of the tumor, since surgical resection causes inflamed tissue where neutrophils are recruited; thereby, neutrophils are highly activated and can spread neutrophil extracellular traps (NETs) containing D-MMSN. D-MMSNs are up-taken by the surrounding glioma cells and, as a result, this maximizes drug bioavailability to eliminate remaining glioma cells [116]. Notably, catalytically active NSPs are not a prerequisite for the formation of NETs, as NSPs are released as inactive proteases [117].

## 6. Conclusions

Immune evasion of tumor cells by down regulation of MHC I molecules activates NK cell function since NK cells are specialized to eliminate tumor cells in an MHC I antigen presentation independent pathway. Therefore, activating and inhibitory receptors expressed on the cell surface of NK cells are responsible and are promising candidates for immunotherapy. Taken together, NK cells harbor tumor cell surveillance tasks and play an important role in the anti-tumor response by interfering with tumor cell-mediated immune evasion. However, tumor cells have developed further strategies to mask their recognition by NK cells. Understanding the mechanisms of immune evasion provides the basics to assess NK cells in immunotherapy and how to potentiate their anti-tumor efficiency.

## Abbreviations

The following abbreviations are used in this manuscript.

ADCC	antibody-dependent cell-mediated cytotoxicity
APCs	antigen-presenting cells
Cat	cathepsin
CatG	cathepsin G
CCL1	chemokine C-C motif ligand 1
CSPG4	chondroitin sulfate proteoglycan 4
DCs	dendritic cells
D-MMSN	doxorubicin-loaded magnetic mesoporous silica nanoparticles
DNAM-1	DNAX accessory molecule 1
DNRII	TGF-β-dominant-negative receptor II
EGFR	epidermal growth factor receptor
EGFRvIII	epidermal growth factor receptor variant III
GM-CSF	granulocyte macrophage colony-stimulating factor
HLA	human leukocyte antigen
IL-2	interleukin 2
ITAM	immunoreceptor tyrosine-based activation motif
KIRs	killer immunoglobulin-like receptors
MDSCs	myeloid-derived suppressor cells
MHC I	major histocompatibility complex class I
NETs	neutrophil extracellular traps
MICA	MHC class I chain-related protein A
MICB	MHC class I chain-related protein B
NCR	natural cytotoxicity receptor
NK cells	natural killer cells
NKG2D	natural killer group 2D activating receptor
NKG2DL	natural killer group 2D ligands
NSPs	neutrophil serine proteases
PBMCs	peripheral blood mononuclear cells
TCR	T cell receptor
TGF-β	transforming growth factor beta
TGN	trans-Golgi network
Th cells	T helper cells
TME	tumor microenvironment
TNF-α	tumor necrosis factor α
TRAIL	TNF-related apoptosis-inducing ligand
Tregs	T regulatory cells
ULBP1–6	UL 16 binding protein 1–6
